# β‑Hairpin Antimicrobial Peptides: Class Diversity and Sequence Analysis

**DOI:** 10.1021/acsinfecdis.5c01055

**Published:** 2026-02-02

**Authors:** Rabina Ramtel, Richard Gu, Mutiat A. Abdulkareem, Justin R. Randall

**Affiliations:** Division of Biological and Biomedical Systems, University of Missouri − Kansas City, Kansas City, Missouri 64110-2446, United States

**Keywords:** β-hairpin, antimicrobial, peptide, cyclic, disulfide, sequence

## Abstract

Interest in antimicrobial peptides has increased dramatically over the last few decades as researchers continue to explore their potential as alternatives to small molecules, as well as their applications in agriculture and food preservation. One promising yet small antimicrobial peptide class is that consisting of a single β-hairpin cyclized via intramolecular disulfide bonds, commonly termed β-hairpin antimicrobial peptides (β-AMPs). Their short length constrained cyclic structure and wide range of activities make them exciting to the general scientific community and drug developers alike; however, despite being found across several phyla, there remain fewer than 30 identified sequence families, making them exceedingly rare relative to more common structural classes. In this review, we identify and describe 27 unique macrocyclic β-AMP sequence families from the literature, with an emphasis on newer and lesser-known families. We then analyze the class’s sequence composition both as a whole and broken down by structural region, finding common characteristics including lengths of 11–25 amino acids, cationic charge, two or more cysteine pairs separated by at least three residues, and strong enrichment for arginine relative to lysine. We then discuss strategies for using these sequence characteristics to help expand the class and improve their relative underrepresentation.

## Graphical abstract

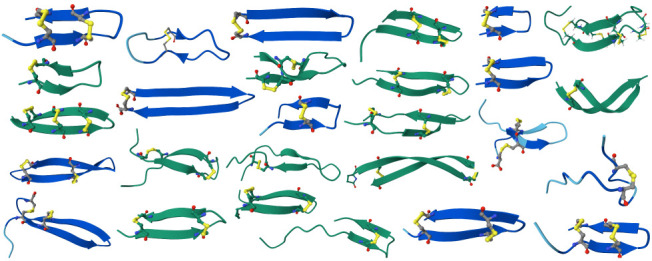

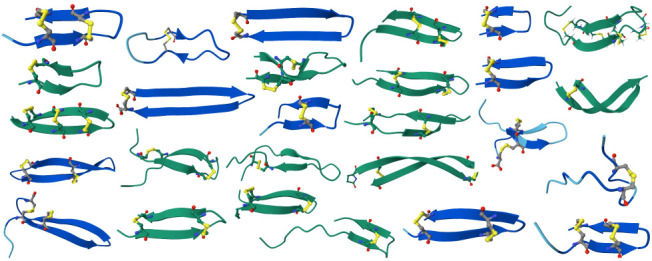

This review focuses on β-hairpin antimicrobial peptides (β-AMPs), defined as short, cationic polypeptides folding into a single pair of antiparallel β-sheets covalently cyclized by one or more intramolecular disulfide bonds^1^ (Figure 1A). This simple, constrained secondary structure limits their length and confers greater proteolytic and thermal stability relative to other antimicrobial peptide classes.^2–4^ We consider β-AMPs to be macrocyclic despite covalent disulfide bonds being reversible, because these covalent bonds are often critical to the stability and activity of the class.

**Figure 1:**
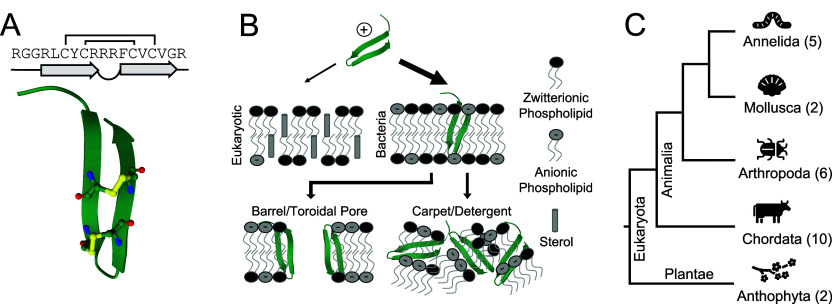
β-AMP structure, membrane activity, and natural phyla distribution. (A) Primary, secondary, and tertiary structures of a typical β-AMP, Protegrin-1 (PDB ID = 1PG1), with cysteine residues involved in disulfide bonds highlighted. (B) β-AMPs often fold into an amphipathic β-hairpin structure in membrane environments. Their cationic charge draws them preferentially to more negatively charged membranes where they then aggregate and disrupt membranes via barrel/toroidal pore or carpet and detergent mechanisms. (C) Tree showing distribution of β-AMP families across different phyla with the number of individual families from each in parentheses.

Like other antimicrobial peptides, β-AMPs commonly adopt amphipathic conformations in membrane or membrane-mimicking environments, meaning they contain spatially separated hydrophilic and hydrophobic regions, allowing them to interact with cell membranes.^5^ Their overall cationic charge draws them preferentially to anionic lipids commonly found in bacterial membranes, like lipopolysaccharide and phosphatidylglycerol, rather than zwitterionic lipids found in eukaryotic membranes.^6,7^ After binding and insertion, they are predicted to destabilize membranes through self-assembly/aggregation into pores (barrel-stave/toroidal) or via a carpet and detergent mechanism, like what has been proposed for other antimicrobial peptides^8–11^ (Figure 1B). A small subset of β-AMPs show alternate or multiple modes of action, which can include inhibition of periplasmic processes, immunomodulatory effects, or other nonlytic activities. These combined attributes have resulted in an extensive study of their therapeutic potential.^12^

To our knowledge, natural identification of β-AMPs has been restricted to the Animalia and Plantae kingdoms in the Eukaryota domain, and they predominantly function biologically as host-defense peptides against microbial pathogens.^1^ We were able to identify only 25 naturally occurring families of β-AMPs from the literature, spread across five phyla: Annelida, Mollusca, Arthropoda, Chordata, and Anthophyta (Figure 1C). Identification of only 25 families, despite significant evolutionary conservation, suggests that many β-AMPs may remain undiscovered. In this review, we aim to explore the β-AMP class’s sequence diversity, with an emphasis on new and synthetic members, and discuss strategies to improve future β-AMP discovery in hopes of increasing their representation among the broader AMP landscape.

## Well-Described Families

Several β-AMPs have been known and well-studied for decades.^1,12^ Most were isolated from cell extracts or biological fluids and commonly occur from proteolytic processing of a larger proprotein. Many also contain high toxicity toward human cells, which continues to complicate their therapeutic use. Here, we provide a brief overview of several families with a description of how they were discovered and their reported antimicrobial activities. A general summary for a single member of each family is provided in Table 1, and an extended table with additional family members can be found in Datafile 1.

**Table 1:**
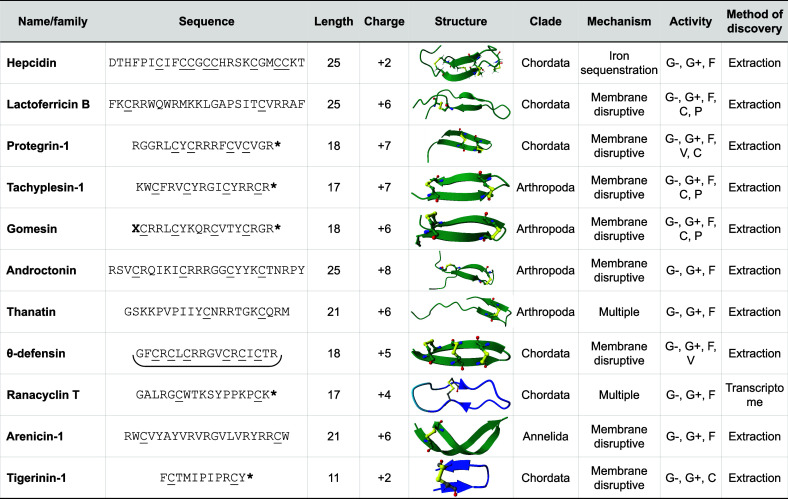
Well-Described β-AMP Families^a^

a G–, Gram-negative bacteria; G+, Gram-positive bacteria; F, fungi; C, cancer; P, parasite; V, viral; *, C-terminal amidation; X, pyroglutamic acid; C, cysteine participating in disulfide bond; green structures are published PDB structures, blue structures are AlphaFold predictions where darker shades have increasing confidence; yellow indicates disulfide bonds; and θ-defensin is cyclized end to end.

### Hepcidins

Hepcidins are a sprawling β-AMP family cyclized via four intramolecular disulfide bonds commonly found in vertebrate animals, more specifically humans and fish.^13,14^ Human hepcidin, also known as liver-expressed AMP-1 or LEAP-1, was originally purified from urine and later also found in blood and the liver.^15^ It shows strong direct antibacterial activity against *E. coli*, with weaker activity against other Gram-negative, Gram-positive, and fungal species.^15^ Hepcidins were also shown to be an important iron regulator, and their antimicrobial activity has been attributed to iron sequestration.^16,17^ Dysfunction in human hepcidin and related genes can cause iron overload diseases, such as hemochromatosis.^18^ Many hepcidins are also expressed in fish gills. One recent example is the identification of DmHep_8cysV1 and DmHep_8cysV2, identified in *Dissostichus mawsoni* and Antarctic fish living in some of the coldest waters in the world.^19^

### Lactoferricin B

Lactoferricin B was originally discovered as a product of pepsin proteolysis of the bovine milk protein lactoferrin.^20^ It is 25 amino acids long and contains a single disulfide bond.^21^ Later identified in humans, Lactoferricin B shows antibacterial, antiviral, antifungal, parasiticidal, and antitumor activities with both a direct membrane disruption mechanism and important immunomodulatory effects.^22–27^ A fragment consisting of the first 11 residues of human lactoferricin (hlF1–11) has been extensively studied in biomaterials and as an antifungal agent.^28,29^

### Protegrins

Protegrins were originally isolated from porcine leukocytes and currently consist of five total family members.^30,31^ All five members are highly similar in sequence and consist of 16–18 amino acids rich in arginine with two intramolecular disulfide bonds and C-terminal amidation.^32,33^ Protegrin-1, the most well-studied member, has broad-spectrum antimicrobial activity targeting a wide range of bacterial, fungal, and viral pathogens through membrane-disruptive pore formation.^34–36^ Protegrin-1 also shows significant toxicity,^37^ which has been associated with both its conformational flexibility and hydrophobicity.^38^ It has also been examined for its anticancer properties and potential amyloid inhibition.^39,40^ Multiple studies have rationally designed Protegrin-1 mimetics/analogues to improve bacterial selectivity,^41,42^ including a recent machine learning-assisted deep mutational sequence analysis reporting how millions of mutations impact its cell specificity.^43^

### Tachyplesins and Polyphemusins

Tachyplesins and Polyphemusins were originally isolated from the lymphatic cells of different species of horseshoe crab.^44–47^ Both families consist of three members (I, II, and III), are 17 or 18 amino acids long, contain two disulfide bridges, and feature C-terminal amidation.^48^ Tachyplesins can exhibit diverse membrane-disruptive activities, including antibacterial, antifungal, antiparasitic, and anticancer effects as well as endotoxin-neutralizing and anti-inflammatory activities.^37,45,49–51^ The most well-studied member of these two families, Tachyplesin I, has been suggested to also inhibit unsaturated fatty acid synthesis and bind double-stranded DNA.^52,53^ Analogues of Tachyplesin I have been studied extensively for their therapeutic potential;^50,54,55^ a recent summary of many of these studies can be found elsewhere.^56^ Interestingly, development of resistance to Tachyplesin I has been observed in *Pseudomonas aeruginosa* and is attributed to efflux pump overexpression and decreased outer membrane permeability.^57,58^

### Gomesin and Androctonin

Gomesin was originally isolated from hemocyte extracts of the tarantula spider *Acanthoscurria gomesiana*.^59^ It was found to be cyclized via two disulfide bonds, contains an N-terminal pyroglutamic acid, and has an amidated C-terminus.^60^ It shows broad activity against bacteria, parasitic protozoa, and fungi. Interestingly, it has also been explored for its antitumor effects, where it has been shown to dysregulate calcium influx.^61,62^ Androctonin was isolated from the hemolymph of the scorpion *Androctonus australis* and similarly contains two disulfide bonds and broad-spectrum activity.^63–65^ Tachyplesins, Polyphemusins, Gomesin, and Androctonin all share mild sequence homology and originate within the Arthropoda phylum.

### Thanatins

Thanatin was originally identified from the hemolymph of the spined soldier bug *Podisus maculiventris*. It consists of a 21-amino acid polypeptide processed from a larger proprotein with a long N-terminal tail cyclized via a single disulfide bond.^66^ Interestingly, Thanatin has been shown to contain multiple mechanisms of action, including membrane disruption, lipopolysaccharide transport inhibition, and New Delhi metallo-β-lactamase-1 inhibition.^66–70^ It preferentially kills *E. coli* and other closely related Gram-negative species, like *Klebsiella pneumoniae*, with far less potent activity also reported against other Gram-negative and Gram-positive bacteria as well as fungi. Several additional family members from the bean bug *Riptortus pedestris* and other related insects have been identified, including the RIP-3 and RIP-4 subfamilies.^71–73^ Thanatin specific reviews examining its multiple modes of action and therapeutic potential in more detail have recently been published.^74,75^

### θ-Defensins

Vertebrate defensins are commonly divided into α-, β-, and θ-Defensins. They share β-sheet regions and three intramolecular disulfide bonds; however, θ-Defensins are the only subfamily with a single β-hairpin secondary structure.^76^ θ-Defensins 1–3 were originally found in leukocyte extracts of the rhesus monkey *Macaca mulatta* and consist of an 18-residue macrocyclic structure stabilized by three disulfide bonds and covalent end-to-end cyclization.^77,78^ They were also later identified in other primate species and have potent antimicrobial activity against bacteria, fungi, and viruses acting through disruption of cell membranes. Closely related retrocyclin pseudogenes have also been identified in humans but are unlikely to be expressed.^79^ Retrocyclins have been explored extensively as antivirals, especially against herpes and HIV.^80^

### Ranacyclins

Ranacyclins are a relatively large family of β-AMPs originally identified in the frog skin secretions of *Ranidae* species.^81^ They are generally 17 to 20 amino acids in length and are cyclized by a single disulfide bond and amidated on their C-terminus. Ranacyclin T and other ranacyclins contain activity against Gram-negative, Gram-positive, and fungal species. Some members of the family contain protease inhibition activity and are considered the smallest members of the Bowman–Birk family of serine proteases.^82^

### Arenicins

Arenicins were originally extracted from the lugworm *Arenicola marina’s* coelomocytes.^83^ The three family members consist of 21-amino acid peptides with either one (Arenicin-1 and -2) or two (Arenecin-3) intramolecular disulfide bonds.^84^ Arenicin-1 and -2 sequences were found to be part of a 202-amino acid precursor proprotein in cDNA race experiments. Arenicins show broad-spectrum antimicrobial activity against bacteria and fungi through membrane disruption.^83,85^ There are many studies looking at Arenicin family therapeutic optimization.^3,86,87^ Arenecin-3 was found to be the least toxic of the family, and analogues have been patented and optimized for better clinical activity, including AA139, which shows promise against multiple antibiotic-resistant pathogens in mouse and rat models of infection.^88–90^

### Tigerinins

Tigerinins are short, macrocyclic β-AMPs isolated in adrenaline-stimulated skin secretions from the frog *Rana tigerina*.^91^ There are four family members (Tigerinin-1,-2,-3,-4), each consisting of 11 or 12 amino acids cyclized via one disulfide bond. They have broad-spectrum antimicrobial activity against bacteria and fungi and function via membrane permeabilization.^91^ Antitumor activity has also been observed for Tigerinin-1,^92^ and a related peptide Tigerinin-1R has been shown to stimulate insulin release.^93,94^

## New and Lesser-Known Families

Over the past decade or so, the number of confirmed macrocyclic β-AMP families has more than doubled, in large part due to the application of new strategies of discovery including -omics mining, protein fragmentation, and high-throughput synthetic screens. Here, we categorize these lesser-known β-AMP sequences by the method of initial discovery and give a brief description of their characteristics and activities. A general summary of each is provided in Table 2. An extended table with additional family members can be found in Datafile 1.

**Table 2:**
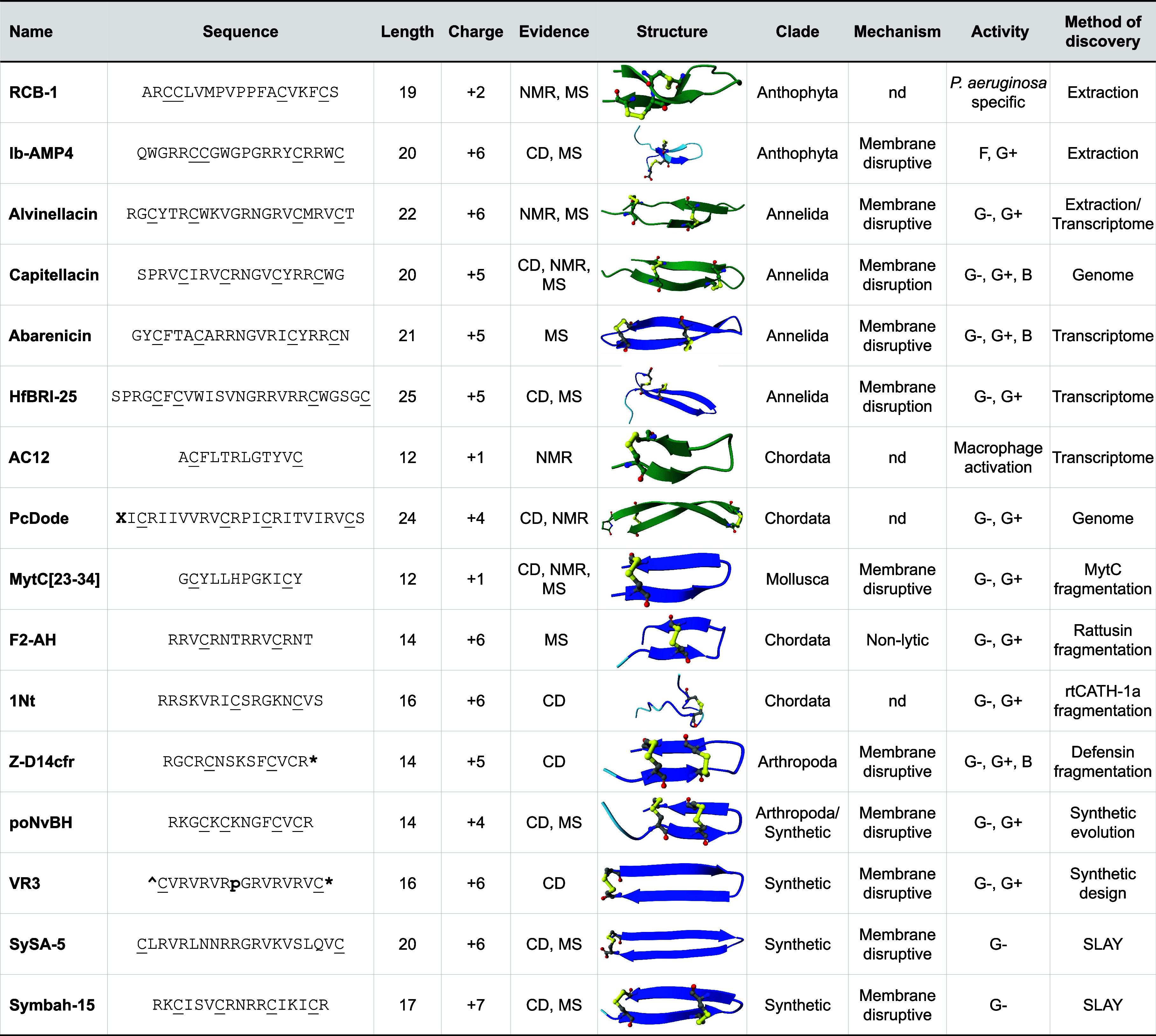
New and Lesser-Known β-AMP Families^a^

a NMR, nuclear magnetic resonance; MS, mass spectrometry; CD, circular dichroism; nd, not determined; G-, Gram-negative bacteria; G+, Gram-positive bacteria; B, biofilm; F, fungi; ^ , N-terminal acetylation; *, C-terminal amidation; X, N-terminal pyroglutamic acid; p, d-proline; C, cysteine participating in disulfide bond; SLAY, surface localized antimicrobial display; green structures are published PDB structures; blue structures are AlphaFold predictions where darker shades have increasing confidence; and yellow shows disulfide bonds.

### Criteria Used for Inclusion in the Class

The criteria used for the inclusion of antimicrobial peptides in the β-hairpin class are not always well-defined. Confirmed structures uploaded to the protein database (PBD) are usually deduced through nuclear magnetic resonance (NMR), but this technique is less frequently applied to lesser-known families. β-Hairpin secondary structure is commonly determined by circular dichroism spectroscopy (CD), where spectra containing minimum/maximum near wavelengths 218/195 correspond to β-sheet secondary structures.^95^ Conformational change from unstructured to β-sheet structures often necessitates membrane-mimicking environments through the inclusion of lipids or detergents in CD buffers. The presence of a disulfide bond is commonly determined by a combination of liquid chromatography and high-resolution mass spectrometry (MS), where formation results in the loss of two hydrogen atoms, corresponding to an equivalent drop in monoisotopic mass. Structural modeling can support β-hairpin structure and the presence of disulfide bonds, but it is important to remember that these models are generally trained on data from much larger and more complex proteins and therefore can be unreliable when predicting the structure of shorter polypeptides. For inclusion in the β-AMP class, we required at least some experimental evidence beyond just structural modeling (Table 2), though a few additional sequences lacking additional experimental evidence are also discussed.

### Extraction

Isolation of antimicrobial peptides through general extraction remains a successful strategy for new discovery. RCB-1, 2, and 3 were extracted from the castor bean *Ricinus communis* and contain two unusual nonconcentric disulfide bonds.^96^ RCB-1’s β-hairpin structure was recently solved by NMR and shows stability in serum for over 24 h.^97^ Uniquely, it seems to show specific activity toward *P. aeruginosa* rather than broad-spectrum antimicrobial activity. Ib-AMP4 and three closely related homologues (Ib-AMP1, 2, and 3) are disulfide-bonded β-AMPs originally extracted from the seeds of the rose balsam *Impatiens balsaminais*.^98^ More recently, Ib-AMP4 was confirmed to have a β-sheet structure in a membrane-like environment via CD. Interestingly, it shows activity against both fungi and Gram-negative bacteria, but not most Gram-positive species tested.^99,100^ RCB-1 and Ib-AMP are the only β-AMP members we could identify originating in plants, both from the *Anthophyta* (flowering plant) phylum. Alvinellacin is the first AMP isolated from a deep-sea organism, extracted from the coelomocytes of the hydrothermal worm *Alvinella pompejana*.^101^ Its mature form is processed from a larger proprotein containing a conserved BRICHOS domain also found in Arenicin proproteins. This BRICHOS domain is associated with chaperone activity, preventing the aggregation of β-rich sequences like β-AMPs.^102^ Alvinellacin is a 22-amino acid peptide cyclized via two disulfide bonds and shows antibacterial activity against both Gram-positive and Gram-negative bacteria via a membrane disruption mechanism.

### Genome and Transcriptome Mining

Advancements in DNA sequencing and computational power have made searching genomes and transcriptomes for potential antimicrobial peptide sequences more easily accessible. This has resulted in the identification of several new β-AMP sequences over the past decade. The most successful strategy thus far has been looking for the previously mentioned BRICHOS domain-containing proproteins, which might be cleaved into macrocyclic β-AMPs. Recently, three additional β-AMPs were identified using this strategy: Capitellacin, Abarenicin, and HfBRI-25. Capitellacin comes from *Capitella teleta* (marine worm) and is a 20-amino acid peptide cyclized via two disulfide bonds.^103^ It is broadly antibacterial and also shows antibiofilm activity, working via a slow membrane disruption mechanism. It shows low toxicity in hemolytic assays, and a synthetic analogue (OMN51) shows potential clinical promise in killing multidrug-resistant *Pseudomonas aeruginosa* isolated from cystic fibrosis patients.^104^ The Abarenicin family consists of five members and was identified in *Abarenicola pacifica*, a lugworm related to *A. marina* from which Arenicins were first discovered.^105^ Abarenicins share some homology with Areneicin-3 and three other BRICHOS identified antimicrobial peptides (bvBRI-21, uuBRI-21, and ucBRI-21) also derived from the spoon worm species. It shows broad antibacterial and antibiofilm activity with mild human toxicity, functioning through general membrane disruption. A screen of several simple analogues was also examined with Ap9 and Ap11 showing reduced toxicity while maintaining most of their antibacterial activity.^105^ HfBRI-25 originated in another marine worm, *Heteromastus filiformis*, for which 13 BRICHOS domain containing proproteins were identified.^106^ HfBRI-25 was the only one with a β-hairpin secondary structure cyclized via disulfide bonds. It was shown to have broad antibacterial activity with very little cytotoxicity and a slow membrane-disruptive mechanism of action. HfBRI-25 was also shown to rescue survival in a mouse model of intraperitoneal infection (IP) with a single dose of 10 mg/kg.^106^

Other peptides identified through transcriptome mining include AC12, a short β-AMP cyclized via a single disulfide bond from the tree *Hypsiboas raniceps*.^107^ AC12 shows no direct antimicrobial activity but instead stimulates macrophage activation in vivo to help reduce inflammation. PcDode was identified as an orthologue of cathelicidins in the genome of *Physeter catodon* (sperm whale).^108^ Its structure was resolved via NMR and was found to have an N-terminal pyroglutamic acid. It is active against both Gram-negative and Gram-positive bacteria with very low levels of hemolysis, but its mechanism of action was not resolved in the study.

### Protein Fragmentation

Another more recent method of macrocyclic β-AMP discovery has been the fragmentation of known proteins or those identified via transcriptomics. A perfect example is a 12-amino acid fragment of a mussel protein MytC (MytC­[23–34]), which was shown to have a β-hairpin structure and a single disulfide bond via CD, NMR, and MS.^109^ MytC[23–34] was active against Gram-negative and Gram-positive bacteria and shown to work through a membrane disruptive mechanism of action, although its cytotoxicity was not determined in the study. Similarly, fragmentation of myticodefensins from another mussel species, *Mytilus coruscus*, resulted in the identification of B3-β.^110^ It has a cyclic β-AMP structure currently only supported by modeling and was synthesized with N-terminal acetylation and C-terminal amidation modifications. It showed selective activity against Gram-negative and Gram-positive bacteria, and the mechanism of action was not determined. F2-AH and F2-PH were recently identified via fragmentation of the rat protein Rattusin.^111^ Their β-hairpin structure is supported via modeling and its cyclization via MS. F2-AH was broadly active against Gram-negative and Gram-positive bacteria. Interestingly, it had only low levels of hemolysis, and its mechanism of action was shown to be nonlytic, but was not further resolved.^111^ 1Nt was identified via the fragmentation of the protein rtCath-1a, one of several cathelicidins found in *Oncorhynchus mykiss* (rainbow trout).^112^ 1Nt demonstrated Gram-negative and Gram-positive antibacterial activity; however, its toxicity and mechanism of action were not determined in the study. Z-d14CFR is a 14-amino-acid β-AMP cyclized by two disulfide bonds and amidated on its C-terminus. It is the result of fragmentation of a defensin from *Zophobas atratus* (darkling beetle).^113^ It functions via membrane disruption and shows broad antibacterial and antibiofilm activity with little to no hemolysis. Several derivatives of Z-d14CFR, including Z­(WR)­2 and Z­(WR)­3, were later produced with healing wound infections in mind.^114^

### Synthetic Identification

Rather than discovering from natural sources, some scientists have identified synthetic macrocyclic β-AMPs through rational design or high-throughput screens. poNvBH was designed initially from a fragment of Navidefensin2–2 found in the parasitoid wasp *Nasonia vitripennis*.^115^ It contains 14 amino acids cyclized via two disulfide bonds. It has a membrane-disruptive mechanism of action with activity against Gram-negative and Gram-positive bacteria and mild hemolytic activity. One example of a fully synthetically designed β-AMPs is VR3.^116^ It contains alternating hydrophobic-charged amino acids in its sheet region to promote the β-hairpin structure. It also has N-acetyl and C-amide modifications and a d-proline in its turn region. It has 16 amino acids and is cyclized via a single disulfide bond between the first and last residues of the sequence. It was found to function via membrane disruption with activity against Gram-negative and Gram-positive bacteria with low hemolytic activity. It was also shown to treat a *T. typhimurium* IP infection in a mouse model in the same study.^116^ A similar alternating of hydrophobic-charged amino acids has been used to promote the β-hairpin structure in other peptide sequences. BTT3, which contains no cysteine residues, was instead cyclized via hydrocarbon stapling at various positions.^117^ Another successful strategy in synthetic β-AMP discovery has been to screen large libraries of peptides for antibacterial activity via a technique called surface localized antimicrobial display (SLAY).^118^ The Symbah family of peptides was originally identified in a SLAY screen of over 800,000 random amino acid sequences.^119^ Symbah-1 was shown to have broad-spectrum antibacterial activity with low hemolysis and treated an IP infection from a multidrug-resistant strain of *A. baumannii* in a mouse model.^4^ Symbah-15, one of 23 optimized variants showing a cyclic β-hairpin structure, consists of 17 amino acids with two disulfide bonds. Over 1000 members of the SySA family of antimicrobial peptides were identified in another SLAY screen of 200,000 unique sequences designed with the cyclic β-hairpin structure in mind.^120^ Characterization of five SySA family members via CD and MS showed that they all had macrocyclic β-hairpin structures. The most potent member, SySA-5, consists of 20 amino acids cyclized via a single disulfide bond and shows activity against Gram-negative bacteria with low levels of hemolytic activity.^120^ A third SLAY screen of a 100,000 peptide library based on β-AMP sequence features identified 14 serum-active peptides (SAPs) containing disulfide bonds; however, their secondary structure was not determined in the study and a synthetically modified lead (SAP-26) appeared to be unstructured in membrane-mimicking environments.^121^

## Sequence Analysis and Concluding Remarks

β-AMP under-representation among the greater AMP landscape remains a bit of a mystery. Why has the recent explosion in computational and machine learning-driven AMP discovery not had more impact on β-AMP expansion? Are they really that rare, or are we just not looking for them correctly? Below, we discuss some of the sequence features common to the β-AMP class and how new collaborations and high-throughput technologies could help exploit these features to uncover the true abundance of this class.

### Sequence Analysis

Examination of the sequences and structures in Tables 1 and 2 identifies several obvious common characteristics for this class. All sequences have a β-hairpin secondary structure, an overall cationic charge between 1 and 8, a length between 11 and 25 amino acids, and at least one cysteine pair participating in a disulfide bond. All cysteine pairs are also separated by at least three other residues. End modifications were not uncommon among the class, with C-terminal amidation occurring in six members and N-terminal acetylation observed in one. Pyroglutamate was the only noncanonical amino acid observed in naturally occurring sequences, found as the first residue in both PcDode and Gomesin. To avoid over-representation in our analysis, we used only a single representative from each family (Tables 1 and 2). A quick charting of charge versus the grand average of hydrophobicity (GRAVY) score shows a wide range of both cationic charge and hydrophobicity with no obvious grouping or correlation between phylum of origin (Figure 2A). We next performed a deeper analysis of the individual amino acid frequencies present in the antiparallel β-sheet, tail, and loop regions of the class (Figure 2B). This analysis shows that cysteine is highly enriched in the β-sheet and, to a lesser extent, in the tail regions. Interestingly, we also observed a greater than 2-fold enrichment for arginine relative to lysine across all three regions. In sheet regions, valine, tryptophan, and isoleucine were all somewhat preferred over other hydrophobic amino acids. The loop regions were enriched with expected amino acids like proline and glycine; however, strong enrichment for more surprising amino acids like arginine and asparagine was also observed. Tail regions saw minor enrichment of glycine in addition to the previously mentioned arginine and cysteine. Other small but noteworthy observations include the relative absence of histidine, glutamine, glutamate and aspartate from all three regions, and the region-specific absence of tryptophan from loop regions and proline from sheet regions. It is important to note that our sequence composition analysis was limited by both the rarity of this class and the use of only a single representative from each family. This limited our conclusions to simple trends rather than statistically significant conclusions.

**Figure 2:**
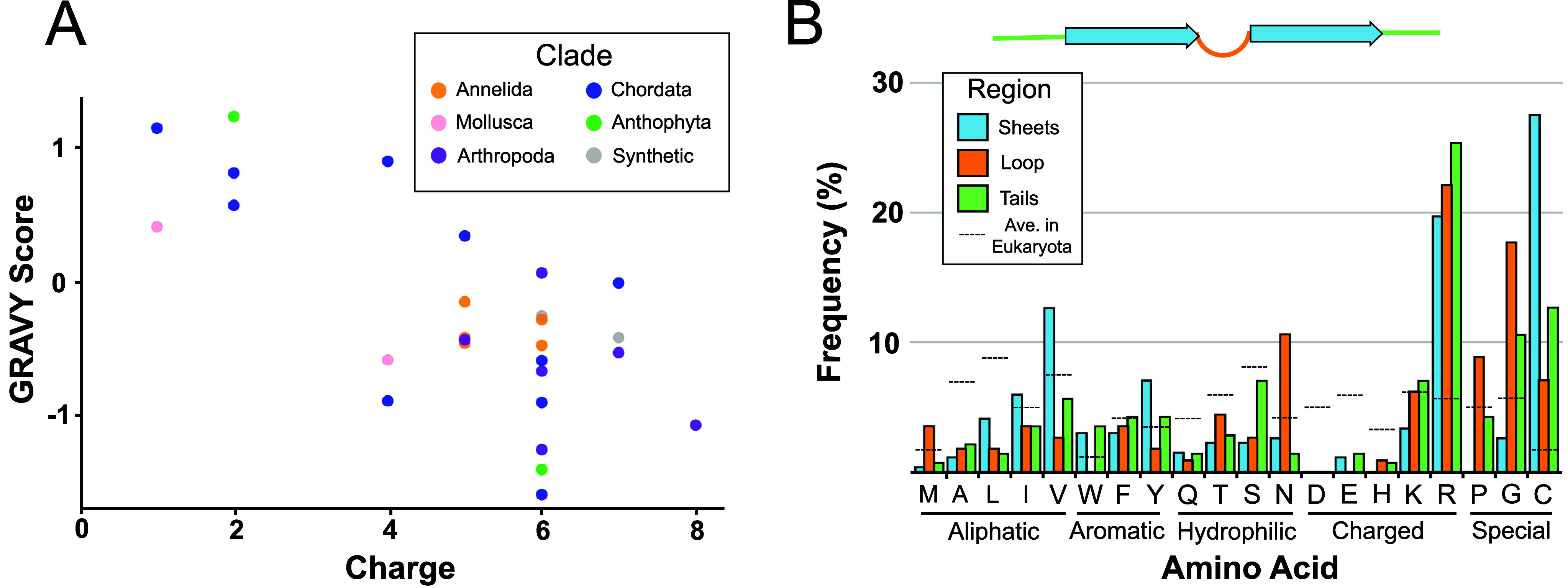
β-AMP sequence analysis. (A) Scatter plot of the charge versus GRAVY score for all β-AMP sequences in Tables 1 and 2 color coded by clade of origin. (B) Amino acid frequencies observed in β-AMP structural regions: antiparallel β-sheets (sheets), loop, and tail. Dotted lines represent the estimated average frequency of each individual amino acid within the Eukaryota domain.^127^

### Concluding Remarks

Our analysis points to multiple sequence characteristics that can be used to identify β-AMPs among large AMP sequence data sets. These characteristics include a length of 11–25 amino acids, cationic charge, two or more cysteine pairs separated by at least three residues, and a strong enrichment of arginine relative to lysine. We can search for these characteristics in current databases containing tens of thousands of known AMPs, like the Database of Antimicrobial Activity and Structure of Peptides,^122^ to help identify new potential β-AMPs. These searches could be paired with structural prediction modeling to further improve the identification of sequences with high β-AMP likelihood whose structure could then be validated with more high-throughput structural assays like CD and MS. Alternatively, large existing data sets of predicted AMPs from high-throughput screens or bioinformatic analysis of -omics data and/or machine learning such as AMPSphere^123^ or proteome-derived antimicrobial peptides^124,125^ could be searched for β-AMP sequence characteristics. Once identified, the antimicrobial activity can be rapidly determined using innovative technologies like SLAY.^119^ Scientists from multiple fields need to collaborate and combine computational biology, machine learning, and high-throughput technologies to help expand and elucidate the true sequence diversity and evolutionary conservation of the β-AMP class.

## Methods

### Figure Visualization

All figures were produced in Microsoft PowerPoint. Tables were made using Microsoft Excel. Graphs were produced using RStudio Version 2025.09 + 418 (2025.09.2 + 418).

### Structural Models

Peptide structures shown in tables and datafiles were created from published PDB structures when available. If no published structures were available, models were produced by entering primary sequences into the AlphaFold 3 server^126^ and visualized using Mol* 3D viewer^127^ available through RCSB PDB (RCSB.org). Any disulfide bonds present in the structures were highlighted. PDB IDs and reference number for each structure are provided in Datafile 1.

### Sequence Analysis

For sequence analysis of β-AMPs, the charge in Figure 2A was calculated by counting the number of positive residues (R, K) and subtracting the number of negatively charged residues (D, E). Additionally, amino and carboxy termini were counted as +1 and −1 unless modified to negate charge (N-terminal acetylation or pyroglutamate; C-terminal amidation). Structure-based charge for β-AMPs with known structure was also calculated using the web-based Adaptive Poisson–Boltzmann Solver (APBS)-PDB 2PQR software suite^128^ (Datafile 1). Published PDB files were put into PDB 2PQR and used as input for the APBS Web server to generate a .pqr file. This file was then used to calculate the surface charge using Python. GRAVY score was calculated by running the primary sequence of each peptide, with end modifications and noncanonical amino acids removed using the “PEPTIDES” R-package (https://cran.r-project.org/web/packages/Peptides/index.html). Specific methods used to calculate hydrophobicity^129^ are detailed in the package. Individual amino acid frequencies were also produced using primary sequences, with end modifications and noncanonical amino acids ignored, where each position was assigned to either tail, sheet, or loop regions according to the corresponding structures in Tables 1 and 2. The full data sets used to generate graphs in Figure 2 are provided in Datafile 1.^130^

## Supplementary Material



## Notes

Datafile 1: Extended table and raw data, including contents (Sheet 1); extended table of β-hairpin antimicrobial peptide families (Sheet 2); and raw data for Figure 2 (Sheet 3) (XLSX)

## Abbreviations Used

β-AMP: β-hairpin antimicrobial peptides
CD: circular dichroism
NMR: nuclear magnetic resonance
MS: mass spectrometry
GRAVY: grand average of hydrophobicity
